# Proton Craniospinal Irradiation for Patients with Solid Tumor Leptomeningeal Disease: Real-World Feasibility, Toxicity, and Outcome Analysis

**DOI:** 10.3390/cancers17061046

**Published:** 2025-03-20

**Authors:** Omer Gal, Alonso La Rosa, Matthew D. Hall, Robert H. Press, Zachary Fellows, Andrew J. Wroe, Alonso N. Gutierrez, Yazmin Odia, Minesh P. Mehta, Rupesh Kotecha

**Affiliations:** 1 Department of Radiation Oncology, Miami Cancer Institute, Baptist Health South Florida, 8900 N Kendall Dr, Miami, FL 33176, USA; omer.gal@baptisthealth.net (O.G.); alonso.larosadelosros@baptisthealth.net (A.L.R.); matthewha@baptisthealth.net (M.D.H.); robert.press@baptisthealth.net (R.H.P.); zachary.fellows@baptisthealth.net (Z.F.); andrewwr@baptisthealth.net (A.J.W.); alonsog@baptisthealth.net (A.N.G.); mineshm@baptisthealth.net (M.P.M.); 2 Herbert Wertheim College of Medicine, Florida International University, 8900 N Kendall Dr, Miami, FL 33176, USA; yazmino@baptisthealth.net; 3 Department of Neuro-Oncology, Miami Cancer Institute, Baptist Health South Florida, 8900 N Kendall Dr, Miami, FL 33176, USA

**Keywords:** leptomeningeal metastases, leptomeningeal disease, craniospinal irradiation, brain neoplasms, proton therapy, molecular factors

## Abstract

**Background/Objectives:** Proton craniospinal irradiation (pCSI) is an emerging treatment option for selected patients with leptomeningeal disease (LMD) that may improve CNS progression-free survival (CNS-PFS) and overall survival (OS) compared to photon involved field irradiation. This study aims to report the applicability of pCSI to standard practice including feasibility, real-world toxicities, and efficacy outcomes. **Methods:** Consecutive patients with LMD from solid tumors who completed intensity-modulated pCSI (30 GyRBE in 10 fractions) at a single tertiary care institution between 2022 and 2024 were included. Toxicity was reported using the Common Terminology Criteria for Adverse Events. OS and CNS-PFS were assessed using the Kaplan–Meier method. Cox regression was used to assess covariates potentially associated with outcomes. **Results:** The cohort included 38 patients with a median age of 58 years (range: 23–82). Most had prior radiotherapy (61%); a reduced dose of 18–25 Gy RBE was delivered to the reirradiated spinal cord in 9 patients (24%). The median time from LMD diagnosis to pCSI completion was 34 days. Concurrent systemic therapy was administered in 10 (26%) patients, most within 30 days before pCSI (58%). Acute grade 3 toxicity comprised lymphopenia (42%); grade 4 toxicity comprised lymphopenia and neutropenia (3% each). The median CNS-PFS and OS were 8.1 and 10.1 months, respectively. Dose discount, systemic treatment, and grade 3+ toxicity were not associated with inferior outcomes. **Conclusions:** pCSI is a safe and feasible treatment option for real-world patients with LMD, including those previously treated with radiotherapy to the spine. Patients in this cohort had minimal interruptions in systemic therapy.

## Simple Summary

We report one of the largest cohorts of patients with leptomeningeal disease treated with modern proton craniospinal irradiation at a large tertiary center. We detail treatment techniques used; including dose reduction applied for previously irradiated spine regions, report cerebrospinal fluid analysis results compared to the primary tumor molecular profile; and outline time intervals for key milestones in the course of treatment. Additionally, we include a full toxicity profile, consisting mainly of lymphopenia, and present efficacy data to compare with the results of the recently published prospective data. Taken together, the detailed information presented in the current manuscript may serve to inform both current practice and future research efforts.

## 1. Introduction

Leptomeningeal disease (LMD) is a devastating clinical scenario in patients with metastatic cancer. The rising incidence of LMD diagnoses in recent years may be attributed to better imaging modalities and more effective systemic therapies controlling extracranial disease [1,2,3,4]. Historically, patients with LMD have a poor prognosis, measured in weeks to months; active treatment, if offered at all, is commonly limited to palliation [5]. Involved-field radiation therapy is limited to the symptomatic region(s) and typically comprises whole-brain and/or focal spinal RT [6]. In-field and out-of-field failures with this approach are common, and overall survival (OS) remains modest [7]. Craniospinal irradiation (CSI) targeting the entire neuroaxis can overcome several of the limitations of involved-field radiation therapy, delaying distant failures and clinical deterioration. However, traditional photon-based CSI is associated with debilitating adverse effects, including nausea, vomiting, xerostomia, fatigue, and severe hematologic toxicities, limiting patient completion and delaying and compromising the intensity of subsequent chemotherapy [8,9,10]. Proton-based CSI (pCSI) presents a novel approach to overcoming these limitations due to favorable organ-sparing properties, improving safety and effectively enabling treatment of the entire neuroaxis [11,12].

Until recently, no prospective data existed to guide treatment for LMD patients. A phase 2 trial reported by Yang et al. randomized 42 and 21 patients to pCSI and photon involved-field radiation therapy, respectively. Meeting its primary endpoint at interim analysis, pCSI improved median CNS progression-free survival (CNS-PFS); secondary endpoints were also met, including improved median OS, with equivalent rates of adverse events [13]. Despite the positive results of this trial, pCSI has not been fully incorporated into international guidelines, with recently reported European Association of Neuro-Oncology LMD-specific guidelines making no recommendation regarding its role [14]. Reluctance in adopting pCSI may arise from trial-related issues, such as the single-institution nature, a highly selected patient population limited to primary non-small cell lung (NSCLC) and breast cancers, exclusion of previously irradiated patients, and withholding of systemic therapy one week before initiation of RT; other reasons include limited availability of pCSI and consequently a lack of data supporting the real-world applicability of the aforementioned trial. In order to bridge this knowledge gap, this study aims to provide evidence for pCSI use in a real-world clinical setting, hypothesizing that pCSI is a feasible, safe, and effective treatment modality in daily clinical practice, and informing current practice as well as future investigations.

## 2. Materials and Methods

### 2.1. Patient Cohort

Patients treated at a single tertiary care institution between January 2022 and August 2024 with pCSI for LMD from solid tumors were queried, following institutional review board approval (protocol number 2024-RETRO-GAL-001). All cases were discussed in a dedicated tumor board, and pCSI was recommended after a multi-disciplinary review of these patients. Patients were eligible if they had unequivocal evidence of LMD on craniospinal MRI and/or positive cerebrospinal fluid (CSF), had completed pCSI treatment, had at least one follow-up visit, and had at least one follow-up neuroaxis MRI assessment for response. Data collected from the electronic medical records included sex, age, laboratory test results, tumor histology, CSF analyses, Karnofsky Performance Status (KPS), prior systemic and radiation therapy, follow-up visits, and MRI studies. Toxicities were reported from treatment start and up to 3 months post-pCSI using Common Terminology Criteria for Adverse Events (CTCAE version 5), as no longer-term toxicity data were available due to the retrospective nature of this study. Radiotherapy treatment dates, dose and fractionation schedules, and detailed treatment plans were available for all patients, including all dosimetric data.

### 2.2. Treatment Planning and Delivery

The pCSI clinical target volume (CTV) was delineated to include the whole brain and spinal canal from C1 to 1.5–2 cm below the thecal sac, also including the proximal spinal nerve roots. The CTV was then expanded by 3 mm for the brain and 5 mm for the spine, to create a planning target volume (PTV) accounting for movement and daily setup variation. The prescription dose was 30 Gy in 10 fractions for all cases. A segmental dose reduction to spinal areas previously exposed to RT was delivered as either 18 Gy in 6 fractions if sequential planning was performed, or 20–25 Gy in 10 fractions if a simultaneous boost technique was utilized, to reach a cumulative equivalent dose in 2 Gy fractions of 60 Gy with an α/β of 3; no dose reduction was made for prior radiosurgery. Institutional standardized pencil beam scanning treatment plans were calculated and robustly optimized with a ±3.5% range and 3–5 mm setup uncertainties in RayStation version 12A SP1 (RaySearch Laboratories AB, Stockholm, Sweden) using the Monte Carlo 5.4 algorithm. The beam arrangement included two posterior-oblique fields for the brain, matched with a 10 cm gradient junction to posterior spine treatment fields. The treatment plans were delivered on an IBA Proteus^®^ PLUS (IBA AB, Louvain-La-Neuve, Belgium) proton therapy system.

### 2.3. Statistical Considerations

Descriptive statistics were used to report patient, tumor, and treatment characteristics. OS and CNS-PFS were assessed using the Kaplan–Meier method. Univariable Cox proportional hazards regression analysis (UVA), denoted as hazard ratios (HRs) with 95% confidence intervals (CIs), was used to assess variables potentially associated with outcomes; statistically significant variables were considered in a multivariable analysis (MVA). The statistical program used was SPSS version 29.0 (Armonk, NY, USA). All statistical analyses were performed using a *p*-value for statistical significance set at <0.05.

## 3. Results

The study cohort included 38 patients. Patient, tumor, and treatment characteristics are detailed in Table 1.

**Figure 1:**
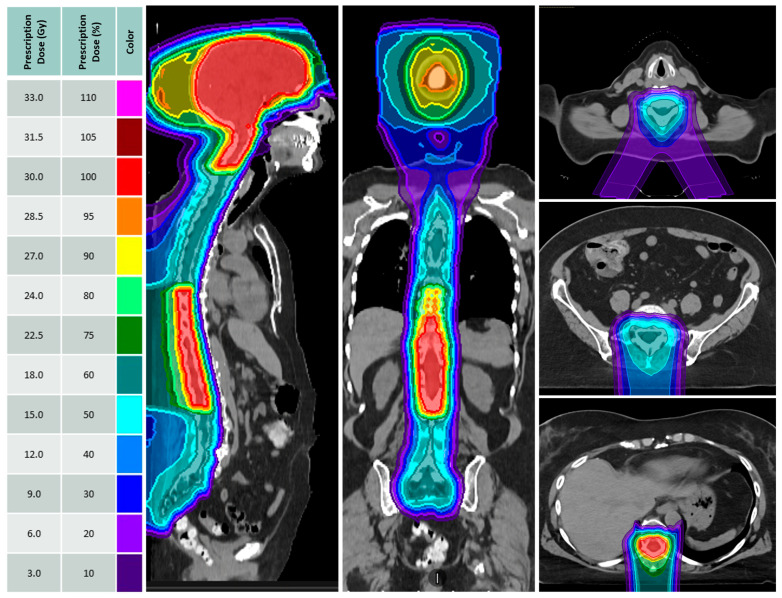
Proton craniospinal irradiation (pCSI) treatment plan with dose discount to previously irradiated spinal regions (18 Gy RBE in 6 fractions sequential plan, teal). Full dose was delivered to the brain and parts of the cervical and thoracic spine (30 Gy RBE in 10 fractions; red).

The median age was 58 years (range: 23–82), 84% were female and 68% were Hispanic. The median KPS was 90 (range 70–100). The most common primary tumor histologies were breast (23, 61%) and NSCLC (10, 26%). Actionable molecular alterations included human epidermal growth factor receptor 2 overexpression (HER2; 9, 24%), epidermal growth factor receptor mutations (EGFR; 4, 11%), and anaplastic lymphoma kinase rearrangement (ALK; 2, 5%). At LMD diagnosis, most patients had extracranial systemic disease (27, 71%). All patients had evidence of LMD on MRI scans involving both the brain and the spine (20, 53%) and mostly occurring synchronously with parenchymal brain metastases (32, 84%). Most patients had prior RT (23, 61%), overlapping with the intended pCSI plan in the brain (14, 37%), spine (3, 8%), or both (6, 16%) regions. Reduced-dose pCSI was delivered to reirradiated spinal regions in 9 patients at a dose of 18–25 Gy RBE in 6–10 fractions, as illustrated in Figure 1; no patients had prior whole-brain radiation therapy.

Concurrent systemic therapy was administered in 10 (26%) patients during pCSI and included osimertinib, pembrolizumab, chemotherapy (2 patients each), afatinib, brigatinib, letrozole and trastuzumab deruxtecan (1 patient each). Most patients received systemic therapy at 30 days or less before pCSI start (22, 58%), with some receiving systemic therapy at 14 days or less before pCSI start (16, 42%).

### 3.1. Feasibility and Toxicity Measures

**Figure 2:**
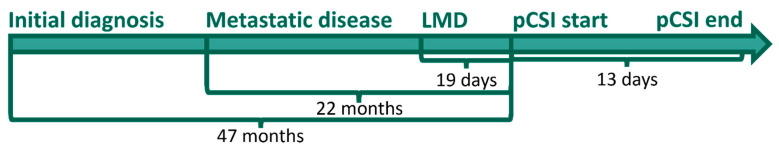
Timeline for milestones in the diagnosis and treatment course of patients treated with proton craniospinal irradiation (pCSI).

Overall, the median time from LMD diagnosis to pCSI completion was 34 days (interquartile range [IQR] 27–53); the median time from LMD diagnosis to CT simulation was 9 days (IQR 5–24), the median time from CT simulation to pCSI start was 8 days (IQR 7–11), and the median time from pCSI start to completion was 13 days (IQR 11–13). Insurance approval was obtained after a median of 1 day (IQR 0–6, Figure 2).

Full toxicity data are detailed in Table 2.

Non-hematologic acute grade 2 toxicity developed in 15 patients (39%), comprising of alopecia (11, 29%), fatigue (6, 16%), anorexia (3, 8%), nausea (2, 5%), and headache and vomiting (1, 3% each); no acute grade 3 or higher non-hematologic adverse events were reported. Grade 2 acute hematologic toxicity was observed in 6 patients (16%), grade 3 toxicity comprising of lymphopenia developed in 16 (42%) patients, and grade 4 toxicity developed in 2 (5%) patients, comprising of lymphopenia and neutropenia (1, 3% each). Late toxicity was reported in 27 (71%) patients and comprised of grade 2 alopecia (3, 11%) and grade 2 and 3 lymphopenia (3, 11% each). Toxicity rates appear similar for patients treated with concurrent systemic therapy compared to patients treated with pCSI alone (Table S1).

### 3.2. Progression-Free Survival and Overall Survival

**Figure 3:**
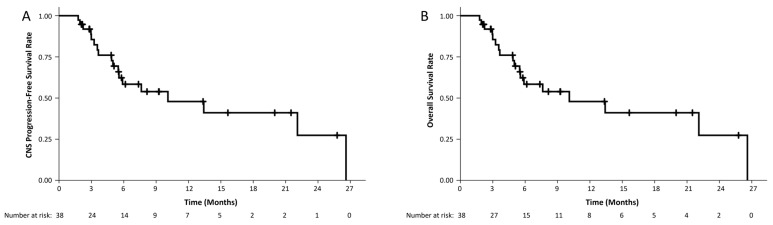
Kaplan Meier curves demonstrating CNS progression-free (**A**) and overall survival (**B**).On UVA, worse OS was associated only with lung versus other non-breast primary tumors (HR 5.76, 95% CI: 1.22–27.09, *p* = 0.03), while on MVA, it was associated only with positive CSF analysis (HR 3.40, 95% CI: 1.03–11.17, *p* = 0.04), as detailed in Table 3. Prior RT, dose reduction, and recent or concurrent administration of systemic therapy were not associated with either CNS-PFS or OS.

The median CNS-PFS was 8.1 months (95% CI: 3.3–12.9), with estimated 6- and 9-month CNS-PFS rates of 53% and 43, respectively (Figure 3A). On UVA, worse CNS-PFS was associated with a lower KPS score (HR 3.08, 95% CI: 1.19–7.94, *p* = 0.02) and lung versus other non-breast primary tumors (HR 5.34, 95% CI: 1.15–24.77, *p* = 0.03). On MVA, increasing age (HR 1.05, 95% CI: 1.01–1.10, *p* = 0.02) and lower KPS scores (HR 3.14, 95% CI: 1.18–8.37, *p* = 0.02) remained statistically significantly associated with CNS-PFS, as detailed in Table 3. Median OS was 10.1 months (95% CI: 1.5–18.7), with estimated 6- and 9-month OS rates of 58% and 53%, respectively (Figure 3B).

### 3.3. CSF Analysis

**Figure 4:**
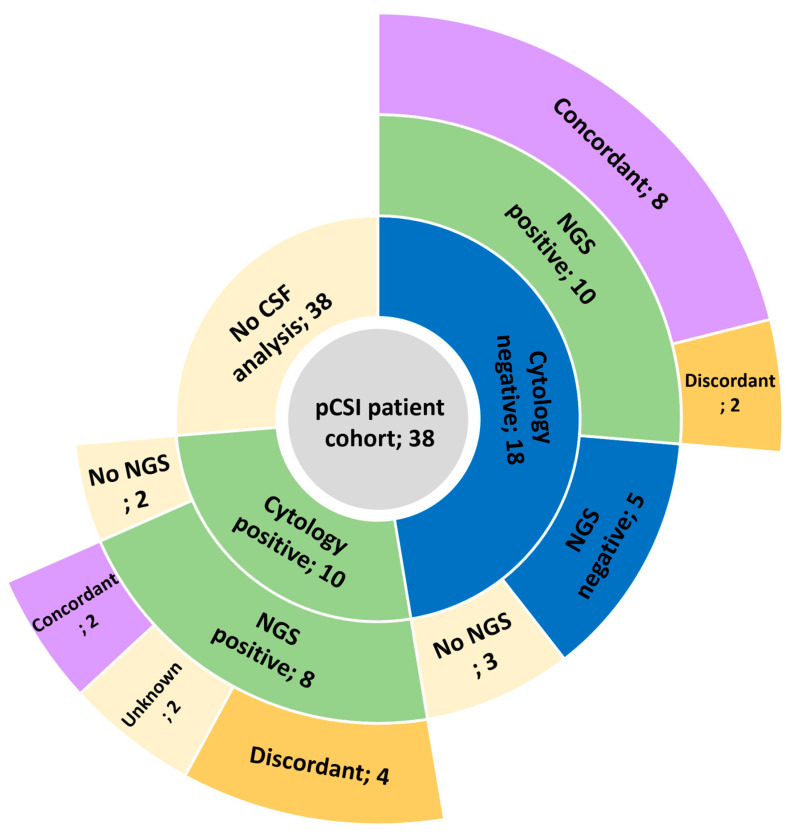
Cerebrospinal fluid (CSF) analysis breakdown for patients treated with proton craniospinal irradiation (pCSI), including cytology, next-generation sequencing (NGS), and concordance with primary tumor molecular analysis.

Ancillary CSF analysis was carried out to verify MRI findings or to obtain further molecular analysis in 28 patients (74%), as illustrated in Figure 4.

CSF cytology was positive for tumor cells in 10 patients (26%), with subsequent next-generation sequencing (NGS) performed in 8 patients, confirming these results. Cytology was negative or inconclusive for 18 patients (48%); however, subsequent NGS performed in 15 detected CSF tumor presence in 10 additional patients, resulting in positive CSF in 20/28 (71%) of the patients tested. Of the 18 positive NGS results, 10 (56%) were concordant with primary tumor molecular alterations, while discordant samples consisted of gain of HER2 amplification in 4 (22%) cases, loss of estrogen receptor (ER) expression in 3 (17%) cases, and loss of EGFR mutation in 1 (6%) case.

## 4. Discussion

LMD continues to represent one of the most devastating manifestations of advanced cancer. This large cohort lends significant support to pCSI as a novel and tolerable treatment option. This study demonstrates the feasibility of pCSI in daily clinical practice for real-life patients of diverse backgrounds (68% Hispanic), while also recapitulating the favorable efficacy and acceptable toxicity reported prospectively.

Incorporation of pCSI into clinical guidelines and standard practice has been partial and slow, despite encouraging results reported in the pivotal randomized phase 2 trial by Yang et al. [13]. Underlying this reluctance were concerns raised regarding the feasibility of delivering timely treatment outside of a clinical trial setting, toxicity, and efficacy in unselected patients [15]. A recent pCSI cohort reported slightly longer intervals, with 16 days from LMD detection to simulation and 12 days from simulation to treatment start, compared to 9 and 8 days in our cohort, respectively [16]. Combining a dedicated team and efficient workflow, this study demonstrates that pCSI can be started expeditiously and generally completed within one month from LMD diagnosis. Additionally, pCSI was recently reported as cost-effective, supporting its incorporation into standard practice [17]. Non-hematologic toxicity was predictable and limited, with no grade 3 or higher toxicity reported. Hematologic toxicity comprised mainly of lymphopenia in 42% of patients during or shortly after treatment, persisting for more than 3 months in 22%. These results are in keeping with previously reported toxicity, for example, grade 3 lymphopenia was reported in 55% by Yang et al. [13]. Importantly, toxicity was not associated with inferior CNS-PFS or OS. We recognize that toxicity should be addressed with caution when reported in hindsight, and limited follow-up time did not allow late adverse events ascertainment in the entire cohort.

In this real-world cohort, both median CNS-PFS and OS were similar to those reported prospectively; in addition, this study recapitulated that positive CSF cytology, age, and KPS were adverse prognostic factors [13]. Importantly, previous RT leading to local dose reduction was not associated with inferior CNS-PFS or OS. Equivalent favorable results were also reported by Lam et al. in a recent publication, although median CNS-PFS and OS were only 3.6 and 4.7 months, respectively, in a preliminary analysis of 17 patients published by the same group [16,18]. Another small pCSI cohort (n = 9) preliminarily reported a short median OS of 4.3 months, highlighting potential biases arising from patient selection and sample size, and emphasizing the value of randomized data [19]. While proton therapy is not yet widely available, and the only existing prospective trial compared pCSI to involved-field radiation therapy; whether similar photon-based CSI can achieve similar outcomes with acceptable toxicity remains unknown. Direct comparison to other cohorts can only be exploratory in nature, yet contemporary photon-based CSI series consistently report short median OS around 3–5 months, not supporting photon-based CSI as an adequate substitute for pCSI [20,21,22,23].

Systemic therapy plays a central role in the treatment of patients with LMD, as most also harbor concurrent extracranial disease [24]. Most patients in this study received systemic therapy during the month preceding pCSI, and concurrent systemic therapy was administered in a quarter of patients in this series. No significant increase in toxicity was observed for patients receiving systemic therapy, suggesting the safety and clinical utility of this approach, though prospective confirmation in a larger cohort is warranted. Moreover, targeting LMD with the same systemic regimen is convenient and potentially less toxic than adding a second modality, and effective for patients with specific molecular alterations common in patients with LMD [25,26,27,28]. However, most patients lack targetable mutations, while others develop LMD while on targeted treatment and/or exhausted effective options. This study also demonstrates discordant molecular alterations in almost half of the available matched primary-CSF samples, in line with other reports [29]. As the therapeutic landscape evolves, upcoming data will help clarify the role and sequence of pCSI and systemic therapy (NCT04588545, NCT06016387) [30].

## 5. Conclusions

In conclusion, this study supports the clinical utility of pCSI as a feasible and safe treatment option for patients with good KPS and LMD arising from NSCLC or breast cancer. Importantly, real-world patients can be treated within a reasonable timeframe, safely reducing the dose for previously treated spine regions, and with concurrent or minimally interrupted systemic therapy. While experience to date is limited to a few small single-center studies and only one prospective trial, the ongoing phase 3 NRG-BN014 trial is expected to provide a definitive answer for the role of pCSI compared to current standard involved-field radiation therapy (NCT06500481).

## Figures and Tables

**Table 1:** Patient, tumor, and treatment characteristics.

Variable	Frequency (n)	Rate (%)
Age, years: median (range)	58 (23–82)	
Sex		
Male	6	16
Female	32	84
KPS		
90–100	20	53
70–80	18	47
Primary tumor histology		
Breast	23	61
HER2+	9	24
NSCLC	10	26
EGFR+	4	11
ALK+	2	5
SCLC	2	5
Other *	3	8
Extracranial disease status		
Active	18	47
Stable/Controlled	9	24
None	11	29
LMD detection method		
MRI	38	100
CSF	20	53
Involved compartment		
Brain	13	34
Spine	5	13
Both	20	53
Parenchymal brain metastases		
Yes	32	84
No	6	16

Abbreviations: KPS = Karnofsky Performance Scale; HER2+ = human epidermal growth factor receptor 2 overexpression; NSCLC = non-small cell lung cancer; EGFR+ = epidermal growth factor receptor mutation; ALK+ = anaplastic lymphoma kinase rearrangement; SCLC = small cell lung cancer; LMD = leptomeningeal disease; MRI = magnetic resonance imaging; CSF = cerebrospinal fluid. * One of each: colorectal, ovarian, and uterine cancers.

**Table 2:** Acute toxicity graded using CTCAE V5.0.

Toxicity/Grade	1	2	3	4
Non-Hematologic: Frequency, n (Rate, %)				
Alopecia	4 (11)	11 (29)	0 (0)	0 (0)
Anorexia	4 (11)	3 (8)	0 (0)	0 (0)
Conjunctivitis	1 (3)	0 (0)	0 (0)	0 (0)
Constipation	2 (5)	0 (0)	0 (0)	0 (0)
Dermatitis	2 (5)	0 (0)	0 (0)	0 (0)
Dizziness	3 (8)	0 (0)	0 (0)	0 (0)
Dry eye	2 (5)	0 (0)	0 (0)	0 (0)
Fatigue	11 (29)	6 (16)	0 (0)	0 (0)
Headache	7 (18)	1 (3)	0 (0)	0 (0)
Memory impairment	2 (5)	0 (0)	0 (0)	0 (0)
Nausea	7 (18)	2 (5)	0 (0)	0 (0)
Seizure	1 (3)	0 (0)	0 (0)	0 (0)
Vomiting	4 (11)	1 (3)	0 (0)	0 (0)
Total	20 (53)	15 (39)	0 (0)	0 (0)
Hematologic: Frequency, n (Rate, %)				
Anemia	0 (0)	2 (5)	0 (0)	0 (0)
Lymphopenia	0 (0)	4 (11)	16 (42)	1 (3)
Neutropenia	0 (0)	1 (3)	0 (0)	1 (3)
Thrombocytopenia	1 (3)	0 (0)	0 (0)	0 (0)
Total	1 (3)	6 (16)	16 (42)	2 (5)

Abbreviations: CTCAE V5.0 = Common Terminology Criteria for Adverse Events version 5.0.

**Table 3:** Univariable and multivariable analysis for CNS-PFS and OS.

Variable		Univariable Analysis		Multivariable Analysis	
	Level	HR (95% CI)	*p*-Value	HR (95% CI)	*p*-Value
PFS					
Age	Continuous	1.03 (0.99–1.07)	0.13	1.05 (1.01–1.10)	0.021
KPS	90–100	REF		REF	
	70–80	3.08 (1.19–7.94)	0.02	3.14 (1.18–8.37)	0.022
Primary tumor	Lung	REF		REF	
	Breast	2.35 (0.68–8.20)	1.79	3.50 (0.90–13.60)	0.071
	Other	5.34 (1.15–24.77)	0.03	4.19 (0.85–20.68)	0.079
OS					
Age	Continuous	1.03 (0.99–1.07)	0.10	1.04 (0.99–1.09)	0.079
KPS	90–100	REF		REF	
	70–80	2.16 (0.82–5.72)	0.12	2.08 (0.68–6.35)	0.199
Primary tumor	Lung	REF		REF	
	Breast	1.47 (0.40–5.34)	0.56	1.18 (0.27–5.06)	0.824
	Other	5.76 (1.22–27.09)	0.03	5.47 (1.00–30.09)	0.051
CSF Analysis	Non-positive	REF		REF	
	Positive	2.65 (0.93–7.56)	0.07	3.40 (1.03–11.17)	0.044

Abbreviations: CNS-PFS = central nervous system progression-free survival; OS = overall survival; KPS = Karnofsky Performance Status; CSF = cerebrospinal fluid; HR = hazard ratio; CI = confidence interval; REF = reference.

## Data Availability

The raw data supporting the conclusions of this article will be made available by the authors upon request.

## Supplementary Materials

The following supporting information can be downloaded at: https://www.mdpi.com/article/10.3390/cancers17061046/s1, Table S1: Acute toxicity according to concurrent systemic therapy according to CTCAE V5.0.

## References

[1] Clarke J.L., Perez H.R., Jacks L.M., Panageas K.S., DeAngelis L.M. (2010). Leptomeningeal metastases in the MRI era. Neurology.

[2] Emoto S., Ishigami H., Yamaguchi H., Yamashita H., Kaisaki S., Kitayama J. (2011). Frequent development of leptomeningeal carcinomatosis in patients with peritoneal dissemination of gastric cancer. Gastric Cancer.

[3] Lai R., Dang C.T., Malkin M.G., Abrey L.E. (2004). The risk of central nervous system metastases after trastuzumab therapy in patients with breast carcinoma. Cancer.

[4] Omuro A.M.P., Kris M.G., Miller V.A., Franceschi E., Shah N., Milton D.T., Abrey L.E. (2005). High incidence of disease recurrence in the brain and leptomeninges in patients with nonsmall cell lung carcinoma after response to gefitinib. Cancer.

[5] Boogerd W., Hart A.A.M., van der Sande J.J., Engelsman E. (1991). Meningeal carcinomatosis in breast cancer. Prognostic factors and influence of treatment. Cancer.

[6] Buszek S.M., Chung C. (2022). Radiation Therapy for Leptomeningeal Disease. Radiopharmaceuticals in the Management of Leptomeningeal Metastasis.

[7] Buszek S.M., Chung C. (2019). Radiotherapy in Leptomeningeal Disease: A Systematic Review of Randomized and Non-randomized Trials. Front. Oncol..

[8] Schiopu S., Habl G., Haefner M., Katayama S., Herfarth K., Debus J., Sterzing F. (2018). Helical tomotherapy in patients with leptomeningeal metastases. Cancer Manag. Res..

[9] Devecka M., Duma M.N., Wilkens J.J., Kampfer S., Borm K.J., Münch S., Straube C., Combs S.E. (2020). Craniospinal irradiation(CSI) in patients with leptomeningeal metastases: Risk-benefit-profile and development of a prognostic score for decision making in the palliative setting. BMC Cancer.

[10] Nguyen T.K., Nguyen E.K., Soliman H. (2021). An overview of leptomeningeal disease. Ann. Palliat. Med..

[11] Yang T.J., Wijetunga N.A., Yamada J., Wolden S., Mehallow M., Goldman D.A., Zhang Z., Young R.J., Kris M.G., Yu H.A. (2021). Clinical trial of proton craniospinal irradiation for leptomeningeal metastases. Neuro-Oncology.

[12] Mathis N.J., Wijetunga N.A., Imber B.S., Pike L.R.G., Yang J.T. (2022). Recent Advances and Applications of Radiation Therapy for Brain Metastases. Curr. Oncol. Rep..

[13] Yang J.T., Wijetunga N.A., Pentsova E., Wolden S., Young R.J., Correa D., Zhang Z., Zheng J., Steckler A., Bucwinska W. (2022). Randomized Phase II Trial of Proton Craniospinal Irradiation Versus Photon Involved-Field Radiotherapy for Patients With Solid Tumor Leptomeningeal Metastasis. J. Clin. Oncol..

[14] Le Rhun E., Weller M., van den Bent M., Brandsma D., Furtner J., Rudà R., Schadendorf D., Seoane J., Tonn J.C., Wesseling P. (2023). Leptomeningeal metastasis from solid tumours: EANO–ESMO Clinical Practice Guideline for diagnosis, treatment and follow-up. ESMO Open.

[15] Wilcox J.A., Chukwueke U.N., Ahn M.-J., Aizer A.A., Bale T.A., Brandsma D., Brastianos P.K., Chang S., Daras M., Forsyth P. (2024). Leptomeningeal metastases from solid tumors: A Society for Neuro-Oncology and American Society of Clinical Oncology consensus review on clinical management and future directions. Neuro-Oncology.

[16] Lam K., Nasr L.F., Andersen C.R., Marqueen K.E., Li J., Wang C., Beckham T.H., Majd N., Aaroe A.E., Loghin M. (2024). Early Outcomes from Proton Craniospinal Irradiation for Leptomeningeal Metastasis from Solid Tumors. Adv. Radiat. Oncol..

[17] Dornisch A., Tringale K.R., Murphy J.D. (2024). Cost-Effectiveness of Proton Craniospinal Irradiation (pCSI) in Patients with Solid Tumor Leptomeningeal Metastasis. Int. J. Radiat. Oncol. Biol. Phys..

[18] Nasr L.F., Li J., Swanson T.A., Ghia A.J., Wang C., Yeboa D.N., Grosshans D.R., McAleer M.F., Beckham T., McGovern S.L. (2023). Early Outcomes from Proton Craniospinal Irradiation (pCSI) for Leptomeningeal Disease from Solid Tumors. Int. J. Radiat. Oncol. Biol. Phys..

[19] Marciscano A.E., Ehret F., Yuan A.M., Zieminski S., Leland P.A., Khandekar M.J., Oh K.S., Shih H.A. (2024). RADT-44. EARLY CLINICAL EXPERIENCE WITH PROTON CRANIOSPINAL IRRADIATION FOR THE TREATMENT OF LEPTOMENINGEAL DISEASE. Neuro-Oncology.

[20] El Shafie R.A., Böhm K., Weber D., Lang K., Schlaich F., Adeberg S., Paul A., Haefner M.F., Katayama S., Sterzing F. (2019). Outcome and prognostic factors following palliative craniospinal irradiation for leptomeningeal carcinomatosis. Cancer Manag. Res..

[21] Takeda K., Umezawa R., Yamamoto T., Takahashi N., Jingu K. (2024). Craniospinal irradiation for leptomeningeal metastasis of solid tumors: Survival analysis and prognostic factors. J. Radiat. Res..

[22] Keit E., Peterson J., Johnstone P.A.S., Robinson T.J., Figura N.B. (2024). Modern Photon-Based Craniospinal Irradiation in Leptomeningeal Disease. Int. J. Radiat. Oncol. Biol. Phys..

[23] Prabhu R.S., Turner B.E., Asher A.L., Marcrom S.R., Fiveash J.B., Foreman P.M., Press R.H., Patel K.R., Curran W.J., Breen W.G. (2019). A multi-institutional analysis of presentation and outcomes for leptomeningeal disease recurrence after surgical resection and radiosurgery for brain metastases. Neuro-Oncology.

[24] Bartsch R., Jerzak K.J., Larrouquere L., Müller V., Le Rhun E. (2024). Pharmacotherapy for leptomeningeal disease in breast cancer. Cancer Treat. Rev..

[25] Yang J.C.H., Kim S.-W., Kim D.-W., Lee J.-S., Cho B.C., Ahn J.-S., Lee D.H., Kim T.M., Goldman J.W., Natale R.B. (2020). Osimertinib in Patients with Epidermal Growth Factor Receptor Mutation-Positive Non-Small-Cell Lung Cancer and Leptomeningeal Metastases: The BLOOM Study. J. Clin. Oncol..

[26] Alder L., Trapani D., Bradbury C., Van Swearingen A.E.D., Tolaney S.M., Khasraw M., Anders C.K., Lascola C.D., Hsu L., Lin N.U. (2023). Durable responses in patients with HER2+ breast cancer and leptomeningeal metastases treated with trastuzumab deruxtecan. npj Breast Cancer.

[27] Sener U., Webb M., Breen W.G., Neth B.J., Laack N.N., Routman D., Brown P.D., Mahajan A., Frechette K., Dudek A.Z. (2024). Proton Craniospinal Irradiation with Immunotherapy in Two Patients with Leptomeningeal Disease from Melanoma. J. Immunother. Precis. Oncol..

[28] Webb M.J., Breen W.G., Laack N.N., Leventakos K., Campian J.L., Sener U. (2023). Proton craniospinal irradiation with bevacizumab and pembrolizumab for leptomeningeal disease: A case report. CNS Oncol..

[29] Wijetunga N.A., Goglia A.G., Weinhold N., Berger M.F., Cislo M., Higginson D.S., Chabot K., Osman A.M., Schaff L., Pentsova E. (2023). Dynamic Mutational Landscape of Cerebrospinal Fluid Circulating Tumor DNA and Predictors of Survival after Proton Craniospinal Irradiation for Leptomeningeal Metastases. Clin. Cancer Res..

[30] Ahmed K., Kumthekar P., Kim Y., DeJesus M., Pina Y., Oliver D., Evernden B., Arrington J., Vogelbaum M., Rosa M. (2024). Octs-07 Trial in Progress: Phase Ii Study of Radiation Therapy Followed by Intrathecal Trastuzumab/Pertuzumab in the Management of Her2+ Breast Leptomeningeal Disease. Neuro-Oncol. Adv..

