# Supplementary material for: Proton Craniospinal Irradiation for Patients with Solid Tumor Leptomeningeal Disease: Real-World Feasibility, Toxicity, and Outcome Analysis
Source: Cancers (Basel). 2025 Mar 20;17(6):1046. doi: 10.3390/cancers17061046 (PMC11940959; doi:10.3390/cancers17061046)
Supplement: Supplementary file 1 [file cancers-17-01046-s001.zip › cancers-3518133-supplementary.pdf]

**Table S1**: Acute toxicity according to concurrent systemic therapy according to CTCAE V5.0								
**Group**	**No Concurrent Therapy (n=28)**				**Concurrent Systemic Therapy (n=10)**			
**Toxicity / Grade**	**1**	**2**	**3**	**4**	**1**	**2**	**3**	**4**
**Non-Hematologic: Frequency, n (Rate, %)**								
Alopecia	3 (11)	8 (29)	0 (0)	0 (0)	1 (10)	3 (30)	0 (0)	0 (0)
Anorexia	2 (7)	2 (7)	0 (0)	0 (0)	2 (20)	1 (10)	0 (0)	0 (0)
Conjunctivitis	1 (4)	0 (0)	0 (0)	0 (0)	0 (0)	0 (0)	0 (0)	0 (0)
Constipation	0 (0)	0 (0)	0 (0)	0 (0)	2 (20)	0 (0)	0 (0)	0 (0)
Dermatitis	1 (4)	0 (0)	0 (0)	0 (0)	1 (10)	0 (0)	0 (0)	0 (0)
Dizziness	1 (4)	0 (0)	0 (0)	0 (0)	2 (20)	0 (0)	0 (0)	0 (0)
Dry eye	1 (4)	0 (0)	0 (0)	0 (0)	1 (10)	0 (0)	0 (0)	0 (0)
Fatigue	9 (32)	2 (7)	0 (0)	0 (0)	2 (20)	4 (40)	0 (0)	0 (0)
Headache	5 (18)	1 (4)	0 (0)	0 (0)	2 (20)	0 (0)	0 (0)	0 (0)
Memory impairment	2 (7)	0 (0)	0 (0)	0 (0)	0 (0)	0 (0)	0 (0)	0 (0)
Nausea	3 (11)	2 (7)	0 (0)	0 (0)	4 (40)	0 (0)	0 (0)	0 (0)
Seizure	1 (4)	0 (0)	0 (0)	0 (0)	0 (0)	0 (0)	0 (0)	0 (0)
Vomiting	2 (7)	1 (4)	0 (0)	0 (0)	2 (20)	0 (0)	0 (0)	0 (0)
**Total**	**14 (50)**	**10 (36)**	**0 (0)**	**0 (0)**	**6 (60)**	**5 (50)**	**0 (0)**	**0 (0)**
**Hematologic: Frequency, n (Rate, %)**								
Anemia	0 (0)	2 (7)	0 (0)	0 (0)	0 (0)	0 (0)	0 (0)	0 (0)
Lymphopenia	0 (0)	4 (14)	13 (46)	0 (0)	0 (0)	0 (0)	3 (30)	1 (10)
Neutropenia	0 (0)	1 (4)	0 (0)	1 (4)	0 (0)	0 (0)	0 (0)	0 (0)
Thormobocytopenia	1 (4)	0 (0)	0 (0)	0 (0)	0 (0)	0 (0)	0 (0)	0 (0)
**Total**	**1 (4)**	**6 (21)**	**13 (46)**	**1 (4)**	**0**	**0**	**3 (30)**	**1 (10)**

Abbreviations: CTCAE V5.0 = Common Terminology Criteria for Adverse Events version 5.0.
